# Radionuclide Small Intestine Imaging

**DOI:** 10.1155/2013/861619

**Published:** 2013-05-30

**Authors:** Jiri Dolezal, Marcela Kopacova

**Affiliations:** ^1^Department of Nuclear Medicine, Charles University in Prague, Faculty of Medicine in Hradec Kralove and Charles University Teaching Hospital, Sokolska 581, 500 05 Hradec Kralove, Czech Republic; ^2^Second Department of Internal Medicine, Charles University in Prague, Faculty of Medicine in Hradec Kralove and Charles University Teaching Hospital, Sokolska 581, 500 05 Hradec Kralove, Czech Republic

## Abstract

The aim of this overview article is to present the current possibilities of radionuclide scintigraphic small intestine imaging. Nuclear medicine has a few methods—scintigraphy with red blood cells labelled by means of ^99m^Tc for detection of the source of bleeding in the small intestine, Meckel's diverticulum scintigraphy for detection of the ectopic gastric mucosa, radionuclide somatostatin receptor imaging for carcinoid, and radionuclide inflammation imaging. Video capsule or deep enteroscopy is the method of choice for detection of most lesions in the small intestine. Small intestine scintigraphies are only a complementary imaging method and can be successful, for example, for the detection of the bleeding site in the small intestine, ectopic gastric mucosa, carcinoid and its metastasis, or inflammation. Radionuclide scintigraphic small intestine imaging is an effective imaging modality in the localisation of small intestine lesions for patients in whom other diagnostic tests have failed to locate any lesions or are not available.

## 1. Introduction

The aim of this paper is to present current possibilities of radionuclide scintigraphic small intestine imaging. Nuclear medicine has a few methods—scintigraphy with red blood cells (RBCs) labelled by means of ^99m^Tc for detection of the source of bleeding in the small intestine, Meckel's diverticulum scintigraphy for detection of the ectopic gastric mucosa, somatostatin receptor scintigraphy for carcinoid imaging, and radionuclide inflammation imaging. Radionuclide scintigraphic small intestine imaging is an effective imaging modality in the localisation of small intestine lesions for patients in whom other diagnostic tests have failed to locate any lesions or are not available. To improve sensitivity, specificity, and location of the area of increased radioactivity abdomen SPECT/CT and PET/CT are recommended. The hybrid SPECT/CT (single-photon emission computed tomography/computed tomography) and PET/CT (positron emission tomography/computed tomography) of the abdomen allow true three-dimensional (3D) image acquisition and display, while at the same time improving the imaging interpretation and accuracy of scintigraphy. Reconstruction of cross-sectional slices uses filtered back or iterative projection.

## 2. Scintigraphy with Radiolabeled Red Blood Cells

Effective and prompt therapy for acute gastrointestinal (GI) bleeding depends on accurate localisation of the site of haemorrhage. Anamnesis and clinical examination can often distinguish upper and lower GI bleeding. Upper GI tract and colon haemorrhage can be confirmed and localised using conventional endoscopy (gastroscopy, push-enteroscopy, and colonoscopy), which is the method of choice. Small intestine bleeding is more problematic and conventional endoscopy has limited value in the small intestine. Innovative but costly small intestine endoscopy methods, capsule enteroscopy, and deep enteroscopy [1, 2] are not readily available in all hospitals. Deep enteroscopy can be defined as the use of an enteroscope to examine the small bowel distal to the ligament of Treitz or proximal to the distal ileum. The term deep enteroscopy includes double-balloon, single-balloon, and spiral enteroscopy [3]. 

 Scintigraphy with red blood cells (RBCs) labelled by means of ^99m^Tc (T 1/2 6 hours) can help to detect the source of GI bleeding in the small intestine in patients with timely anamnesis of bleeding in the lower GI tract from an uncertain source (obscure-overt bleeding), melaena and/or haematochezia and improves disease management. Scintigraphy with RBCs can identify the site of bleeding at the rate of 0.1 mL per minute or more [4]. Only 2 to 3 mL of extravasated blood is necessary for detection. This compares favourably with the ability of contrast angiography to detect bleeding rates of about 1 mL/min or greater [5]. 

 In vivo or in vitro radionuclide labelling of RBCs can be used. In vivo method—stannous chloride (10–20 *μ*g/kg) from a commercial pyrophosphate kit is injected intravenously. Tin (2^+^) breaks through the red blood cell membrane and attaches itself to the beta chain haemoglobin to be ready to act as a reducing agent when ^99m^Tc-pertechnetate disodium gets into the cell. After 15–30 minutes ^99m^Tc-pertechnetate disodium in saline solution 0.9% is injected intravenously. The pertechnetate diffuses across the RBCs membrane, where it is reduced by the stannous ions administered previously. In vitro method—blood is first taken from the patient and added to a vial containing stannous chloride. The stannous ion diffuses across the RBC membrane and binds to the haemoglobin. Radioactive labelling is then accomplished by adding ^99m^Tc-pertechnetate, which crosses the RBC membrane and is reduced by stannous ion in the cell. After this the labelled RBCs are reinjected. The in vitro method is preferable because of its superior labelling efficiency, yet on the contrary the in vivo method is simple [6]. 

**Figure 1:**
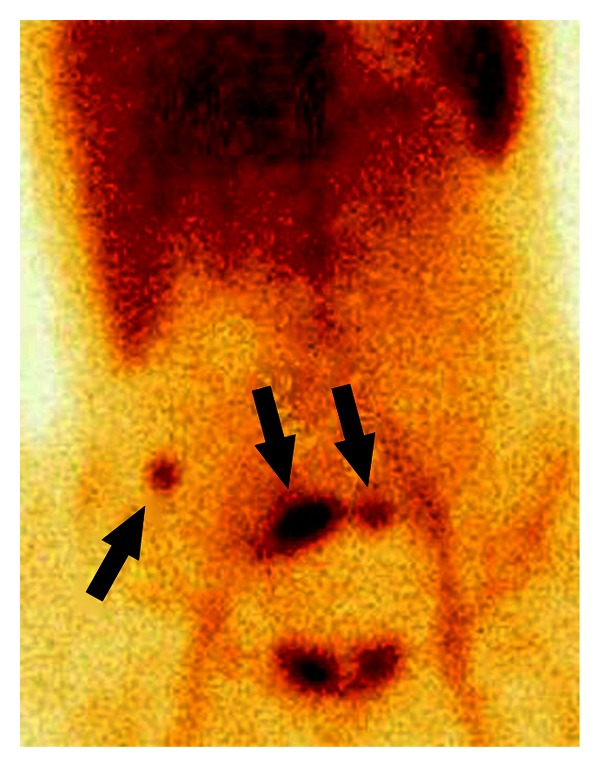
18-year-old woman with enterorrhagia. ^99m^Tc-RBC scintigraphy, anterior view, 15 minutes after intravenous injection of the radiotracer. Bleeding from the ileosigmoidanastomosis six days after hemicolectomy for Crohn's disease (*arrow head*).

**Figure 2:**
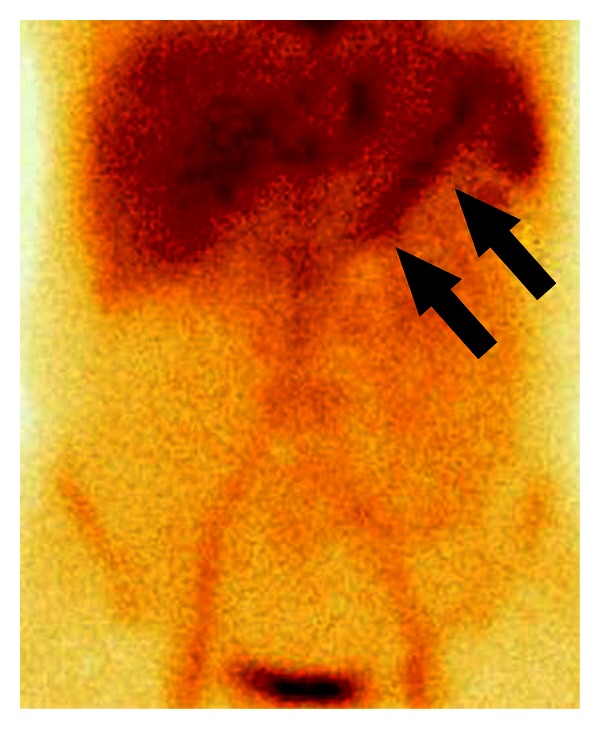
52-year-old man with enterorrhagia. ^99m^Tc-RBC scintigraphy, anterior view, 15 minutes after intravenous injection of the radiotracer. Bleeding at the site of an A-V malformation in the proximal jejunum (*arrow head*).

 Extravasated radiolabeled RBCs within the small intestine are identified as an area of activity that increases in intensity with time, and/or as a focus of activity that moves in a pattern corresponding to the lumen of the small intestine (Figures 1 and 2). Small intestine bleeding can usually be distinguished from colonic bleeding by its rapid serpiginous movement. Steady scintigraphic activity should not be diagnosed as an active bleeding site and usually results from a fixed vascular structure (e.g., haemangioma, accessory spleen, and ectopic kidney) [7].

 In the next part of this chapter we would like to present a few sentences about our experience with the detection of the source of bleeding in the small intestine by means of scintigraphy with in vivo labelled red blood cells (RBCs) in the period of 1998–2012. A fifteen-year prospective study was comprised of 44 patients (27 men, 17 women, aged 12–91, mean 56 years of age) who had gastrointestinal bleeding (obscure-overt bleeding) and underwent scintigraphy with in vivo tagged autological RBCs by means of ^99m^Tc. Scintigraphy was usually performed after other diagnostic tests had failed to locate the bleeding. A total of 28 patients had a positive scintigraphy with in vivo labelled RBCs and 16 patients had negative scintigraphy. The final diagnosis was confirmed in 22 out of 28 patients with positive scintigraphy by push-enteroscopy (7/22), intraoperative enteroscopy (7/22), surgery (4/22), duodenoscopy (1/22), double-balloon enteroscopy (1/22), and X-ray angiography (2/22). The correct location of the bleeding site was identified by RBC scintigraphy in 17 out of 22 (77%) patients with the confirmed source. RBC scintigraphy is an effective imaging modality in localising lower GI bleeding in patients for whom other diagnostic tests have failed to locate the bleeding. RBC scintigraphy can be successful in the detection of bleeding sites in the small intestine.

## 3. Meckel's Diverticulum Scintigraphy

In 1809, the German anatomist Johann Friedrich Meckel Jr. described a “diverticulum” as persistence of the intra-abdominal portion of the vitelline duct, arising from the antimesenteric border of the ileum [8, 9]. Meckel's diverticulum is a true diverticulum containing all layers of the normal intestine wall [10]. The diverticulum arises from persistent ductus omphaloentericus. Meckel's diverticulum is one of the most common congenital anomalies of the gastrointestinal tract [5]. The diverticulum ranges in size from 1 to 11 cm and is located 40–130 cm from the ileocecal valve. It is found approximately 2% of the population [11]. Most commonly, Meckel's diverticulum is asymptomatic, discovered incidentally during surgery [12]. Part of the diverticulum contains ectopic gastric mucosa. Gastric mucosal secretions can cause peptic ulceration, resulting in pain, bleeding, and perforation. About 60% of patients with complications of Meckel's diverticulum are under the age of 2. Bleeding from Meckel's diverticulum after the age of 40 is unusual. Bleeding accounts for most cases. Invagination, intussusception, strangulation by fibrous band, diverticulitis, and perforation are other complications. 

 The indication for Meckel's diverticulum scintigraphy is to localise ectopic gastric mucosa in a Meckel's diverticulum as the source of unexplained gastrointestinal bleeding. ^99m^Tc-pertechnetate disodium is used for imaging gastric mucosa and the uptake is localised to the mucin cell. 

**Figure 3:**
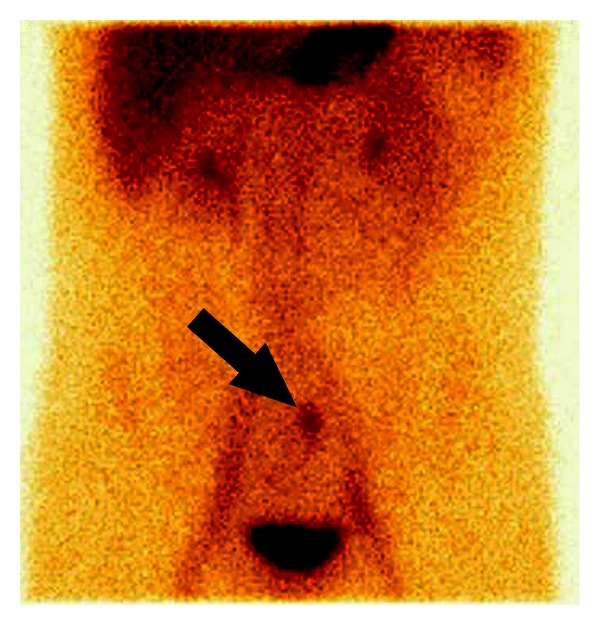
24-year-old man with enterorrhagia. Meckel's diverticulum scintigraphy, anterior view, 20 minutes after intravenous injection of ^99m^Tc-pertechnetate disodium. Radiotracer uptake in the ectopic gastric mucosa in the left hypogastrium (*arrow head*).

 Positive Meckel's diverticulum scintigraphy should have the following subsequent interpretation criteria: (1) radioactivity in ectopic gastric mucosa should appear at the same time as activity in the normal gastric mucosa; (2) a Meckel's diverticulum may appear anywhere within the abdomen, although it is typically seen in the right lower quadrant (Figure 3) [5, 13]. 

 The sensitivity and specificity of Meckel's diverticulum scintigraphy can be improved by pentagastrin, glucagon, and H_2_ blocker. Pentagastrin increases the metabolism of mucus-producing cells, and glucagon inhibits peristaltic dilution and washout of the radionuclide. The pentagastrin test is not commonly used because of the risk of inducing a peptic ulcer in the diverticulum. H_2_ blocker decreases peptic secretion but not radionuclide uptake, retarding the release of ^99m^Tc-pertechnetate from the mucoid cells to the diverticulum lumen [14, 15]. 

 In the next part of this chapter we would like to present our experiences with detection of ectopic gastric mucosa by means of Meckel's diverticulum scintigraphy in patients with lower gastrointestinal bleeding. A total of 139 patients—36 adults (26 males, 10 females, aged 18–78, mean 25 years of age) and 103 children (56 boys, 47 girls, aged 1–17, mean 8 years of age) underwent Meckel's diverticulum scintigraphy for melaena or hematochezia in the period 1994–2012. Scintigraphy was usually performed after other diagnostic tests had failed to locate the bleeding. Six patients (three boys, one girl, and two young men) had positive scintigraphy. All patients underwent surgery and Meckel's diverticulum with ectopic gastric mucosa (by histology) was proven. Meckel's diverticulum scintigraphy can help to detect ectopic gastric mucosa in the abdomen and improve management of patients with lower gastrointestinal bleeding.

## 4. Somatostatin Receptor Imaging

**Figure 4:**
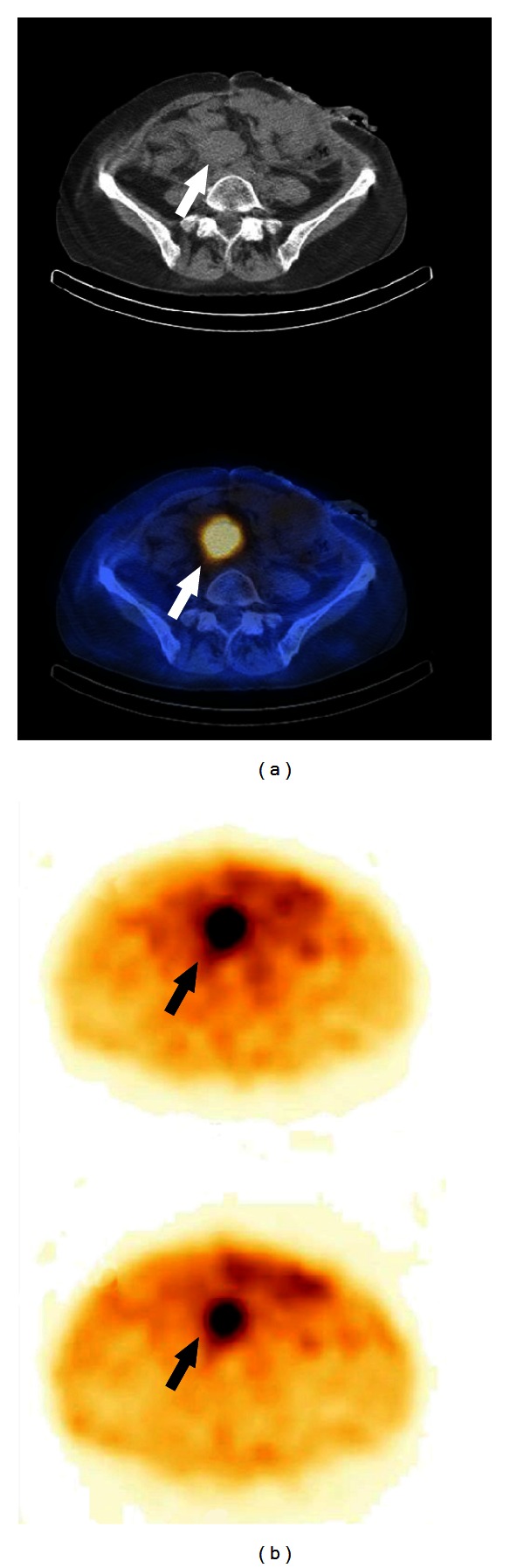
56-year-old woman after ileum resection for carcinoid 15 months ago. Somatostatin receptor imaging with ^111^In-pentetreotide, abdomen CT, SPECT and SPECT/CT, transversal slices. Metastasis of carcinoid in the preaortal lymph node (*arrow head*).

For somatostatin tumour receptor (SSTR) imaging of the carcinoid and its metastases scintigraphy with somatostatin analogue pentetreotid radiolabelled by means of ^11^In (T 1/2 2.8 days) or ^99m^Tc-HYNIC-Tyr^3^-octreotide can be used. Whole body scintigraphy and abdomen SPECT or SPECT/CT are performed (Figure 4). Another possibility for carcinoid molecular imaging is using whole-body PET/CT (positron emission tomography/computed tomography) with somatostatin analogues as ^68^Ga-DOTA-NOC (T 1/2 70 minutes; ^68^Ga-DOTA-1-NaI^3^-octreotide) or ^18^F-DOPA (T 1/2 109 minutes; fluoro-3,4-dihydroxyphenylalanine) [7, 16–18]. Hybrid SPECT/CT or PET/CT allows for three-dimensional (3D) image acquisition and display as well as improved image interpretation.

## 5. Radionuclide Inflammation Imaging

**Figure 5:**
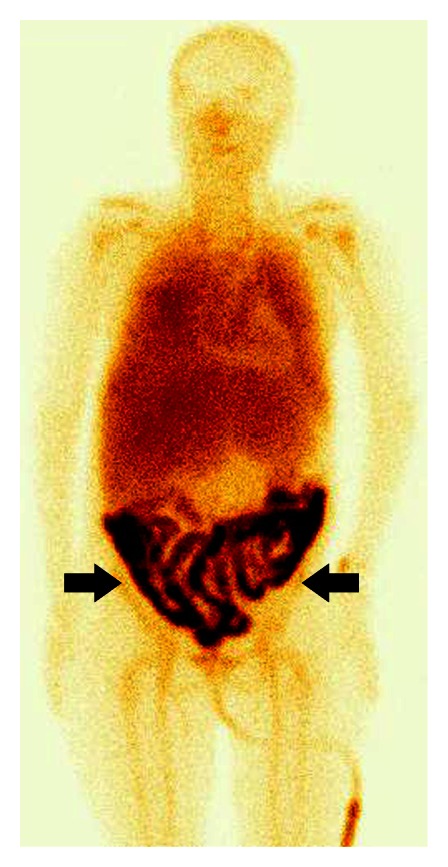
53-year-old woman with fever of unknown origin without abdomen pain. Whole body inflammation imaging with patient leukocytes labelled by means of ^99m^Tc-HMPAO, anterior view. Massive leukocyte uptake in the whole small intestine—m. Crohn.

**Figure 6:**
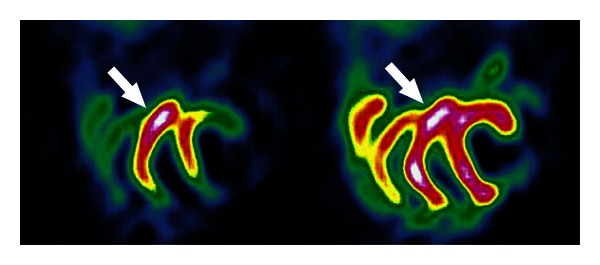
The same patient, abdomen SPECT, coronal slices. Massive leukocyte uptake in the whole small intestine—m. Crohn.

**Figure 7:**
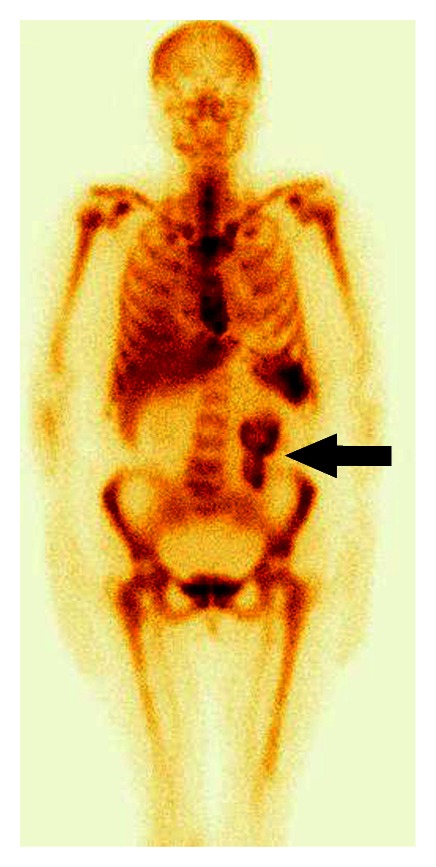
57-year-old woman with fever of unknown origin without abdomen pain. Whole body inflammation imaging with ^99m^Tc-labeled antigranulocyte monoclonal antibodies sulesomab, anterior view. The granulocytes uptake in the ileum—m. Crohn (*arrow head*). Normal uptake of the radiotracer in bone marrow.

**Figure 8:**
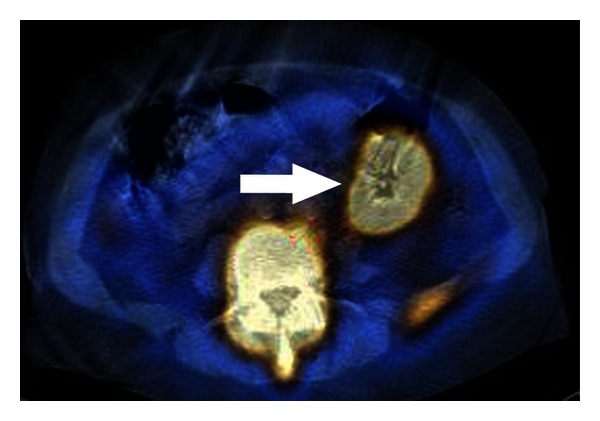
The same patient, abdomen SPECT/CT, transversal slice. Granulocyte uptake in the ileum—m. Crohn (*arrow head*). Normal uptake of the radiotracer in bone marrow.

In a patient with fever of unknown origin radionuclide inflammation imaging can be used when other diagnostic tests have failed to locate anything or are not available. Nuclear medicine has a few radiopharmaceuticals for inflammation imaging, namely, patient leukocytes (Figures 5 and 6) in vitro labelled by ^99m^Tc-hexamethylpropylene amine (HMPAO) or in vivo ^99m^Tc-labeled antigranulocyte monoclonal antibodies (MoAb). This monoclonal antibody sulesomab is directed against a specific granulocyte cell-surface antigen, which is to be found on the surface of granulocytes (Figures 7 and 8). 

 Another possibility for inflammation imaging is ^67^Ga-citrate scintigraphy which still plays an important role in detection and posttreatment evaluation of inflammation. After injection to a peripheral vein, ^67^Ga is bound to the transporting protein transferrin. ^67^Ga is concentrated at the site of inflammation by binding to lactoferrin since ^67^Ga has a higher affinity for lactoferrin from leukocytes than for transferrin. 

 The method for imaging of glucose metabolism in inflammation is positron emission tomography (PET) or hybrid PET/CT. PET with ^18^F-FDG (fluorodeoxyglucose) is useful for inflammation imaging especially in a patient with fever of unknown origin. ^18^F is a positron emitter (half-time 109 minutes). Mechanism of uptake of ^18^F-FDG in inflammation: ^18^F-FDG is phosphorylated in activated leukocytes at the inflammation site by hexokinase to deoxyglucose-6-phosphate, which is not metabolised further and cannot diffuse from leukocytes. A characteristic of inflammation is enhanced glucose metabolism and uptake of ^18^F-FDG. Localisation of ^18^F-FDG at the small intestine inflammation site occurs over the course of a few minutes. By 35 minutes after injection, 95% of peak uptake is achieved [7, 19, 20]. 

## 6. Discussion

Video capsule or deep enteroscopy is the method of choice for detection of most lesions in the small intestine. Small intestine radionuclide imaging is only a complementary imaging methods and can be successful, for example, for the detection of the bleeding site in the small intestine, ectopic gastric mucosa, or primary carcinoid and its metastasis, and so forth, for patients in whom other diagnostic tests have failed to locate anything or are not available. Diehl et al. [21] evaluated the clinical use of RBC scintigraphy in patients with acute lower GI bleeding and negative endoscopy and multislice computed tomography (MSCT). In 31 patients with acute lower GI bleeding in whom the endoscopy findings were negative or the procedure was not feasible, dual-phase MSCT of the abdomen was performed. MSCT was followed by RBC scintigraphy for patients in whom no active bleeding was visible using MSCT. In 20 out of 31 patients MSCT showed no active bleeding and RBC scintigraphy was performed. In 8 of these 20 patients RBC scintigraphy was also negative. In 12 of the 20 patients active bleeding was demonstrated using RBC scintigraphy. Eight of the 12 patients with positive RBC scintigraphy findings underwent surgery, where the site of bleeding was confirmed. Schillaci et al. [22] presented 27 patients with acute lower GI bleeding who were studied with dynamic and planar ^99m^Tc-RBC scintigraphy. In 19 patients with positive scan an abdomen SPECT/CT was performed. In 7 patients SPECT/CT had a significant impact on scintigraphic results and in 6 patients it precisely localised the bleeding foci. Poulsen and Qvist [23] retrospectively evaluated a total of 55 Meckel's diverticulum scintigraphies in 53 patients in comparison with the results of surgery and other diagnostic procedures. Four children had positive scintigraphy. Three patients underwent a laparotomy and Meckel's diverticulum was found. Rampin et al. [24] evaluated the usefulness of Meckel's diverticulum scintigraphy in 28 patients (11 females, 17 males). Scintigraphy was positive in 10 cases and the presence of ectopic gastric mucosa was confirmed by histology. Mittal et al. [25] conducted a retrospective analysis of scintigraphic data of 107 paediatric patients with a suspicion of Meckel's diverticulum. Twenty-one cases from the 107 were positive for functioning gastric tissue indicating Meckel's diverticulum. Two patients were lost to followup and hence surgery could not be performed. The remaining 19 cases were subjected to surgical intervention and 16 were found to be positive for Meckel's diverticulum. Scintigraphy was true positive for ectopic gastric mucosa in 84%. An interesting case report of an adolescent male patient with rectal bleeding and suspected Meckel's diverticulum where the use of SPECT/CT fusion imaging provided valuable diagnostic information and prevented a false-negative study was presented by Dillman et al. [26].

## 7. Conclusion

Radionuclide small intestine imaging is an effective imaging modality in the localisation of the small intestine lesions for patients in whom other diagnostic tests have failed to locate anything or are not available.
